# Exercise rehabilitation in cardiovascular-kidney-metabolic syndrome: a narrative review

**DOI:** 10.3389/fcvm.2026.1735431

**Published:** 2026-03-27

**Authors:** Min Sun, Zhou Yang, Shijun Xu, Hongyang Liu

**Affiliations:** 1 College of Health-Preservation and Wellness, Dalian Medical University, Dalian, China; 2 Department of Heart Intensive Care Unit, The First Affiliated Hospital of Dalian Medical University, Dalian, China

**Keywords:** cardiovascular disease, cardiovascular-kidney-metabolic syndrome, chronic kidney disease, exercise rehabilitation, individualized exercise prescription, type 2 diabetes

## Abstract

Cardiovascular-Kidney-Metabolic Syndrome (CKM) is a multi-organ dysfunction syndrome driven by the interaction of metabolic risk factors, chronic kidney disease, and cardiovascular disease, culminating in adverse cardiovascular events. Exercise rehabilitation is an effective non-pharmacological intervention that retards CKM progression via multi-target mechanisms. This review aims to summarize the mechanisms of exercise rehabilitation in CKM, the research progress on each component, and explore current challenges and future directions, thereby providing a theoretical basis for the comprehensive management of CKM.

## 1 Introduction

In 2023, the American Heart Association (AHA) introduced CKM to characterize the pathophysiological interactions among obesity, type 2 diabetes (T2DM), cardiovascular disease (CVD), and chronic kidney disease (CKD). Affecting over 25% of American adults and 25%–30% of the global population, this multimorbidity comprises five stages with escalating mortality risk: stages 0–1 (metabolic dysfunction without significant target organ impairment), stages 2–3 (early cardiovascular and kidney injury), and stage 4 (severe cardiovascular events or kidney failure) (1, 2). As a non-pharmacological intervention, exercise rehabilitation demonstrates significant clinical value by improving metabolic parameters (blood pressure, lipids, and insulin sensitivity), enhancing cardiovascular function, and modulating kidney function through multi-target mechanisms (3–5). For stages 0–3, exercise rehabilitation focuses on preventing progression through weight management, metabolic control, and intensive monitoring of subclinical CVD and CKD. For stage 4, priorities shift to preserving physical function, minimizing complications, and optimizing quality of life (2, 6, 7).

For this review, we searched PubMed, Embase, Web of Science, Cochrane Library, CNKI, and Wanfang Data up from January 2010 to December 2025. Given the limited direct evidence for CKM, we included high-quality studies examining exercise interventions in CKM as well as its individual components (cardiovascular disease, chronic kidney disease, and metabolic syndrome) to infer mechanisms and clinical applications (Supplementary Figure S1).

## 2 Exercise improves pathophysiological mechanisms of CKM

**Figure 1:**
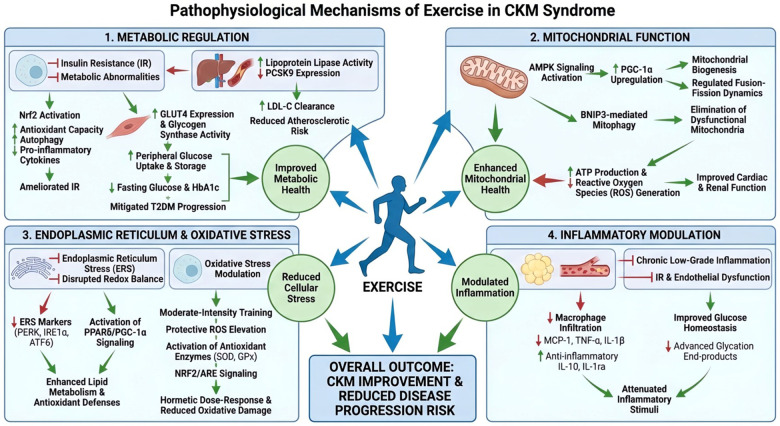
The pathophysiological mechanisms of exercise in CKM syndrome (7, 8). AMPK, AMP-activated protein kinase; ATF6, Activating Transcription Factor 6; BNIP3, BCL2/adenovirus E1B 19 kDa interacting protein 3; GPX, glutathione peroxidase; HO-1, heme oxygenase-1; IL-1β, interleukin-1β; IL-6, interleukin-6; IL-10, interleukin-10; IRE1α, inositol-requiring enzyme 1 alpha; KEAP1, Kelch-like ECH-associated protein 1; MCP-1, monocyte chemoattractant protein-1; NRF2, nuclear factor erythroid 2-related factor 2; PGC-1α, peroxisome proliferator-activated receptor gamma coactivator-1α; PPARs, peroxisome proliferator-activated receptors; SOD, superoxide dismutase; TNF-α, tumor necrosis factor-α.

CKM typically arises from adipose tissue excess, adipose tissue dysfunction, or a combination thereof. These abnormalities trigger chronic inflammation, mitochondrial dysfunction, oxidative stress, and insulin resistance, collectively driving metabolic dysregulation, kidney disease progression, cardio-renal crosstalk, and cardiovascular complications. Exercise intervenes through multiple pathways to delay CKM progression (2, 8, 9) (Figure 1).

### 2.1 Metabolic regulation

Insulin resistance (IR) is a key factor in the metabolic abnormalities of CKM (2). Exercise attenuates IR primarily through Nrf2 activation, which enhances antioxidant enzyme activity, maintains iron homeostasis, promotes autophagy, and suppresses pro-inflammatory signaling (10). Exercise also increases GLUT4 protein expression and glycogen synthase activity, effectively accelerates glucose uptake and glycogen storage, and significantly improves peripheral tissue insulin sensitivity (11). Collectively, these adaptations optimized glucose homeostasis, significantly lowering fasting glucose and HbA1c levels, and significantly reduced T2DM risk. Concurrently, exercise upregulates lipoprotein lipase (LPL) activity to enhance triglyceride (TG) catabolism and downregulates proprotein convertase subtilisin/kexin type 9 (PCSK9) to accelerate hepatic LDL-cholesterol clearance, thereby mitigating atherosclerosis and cardiovascular risks (12, 13).

### 2.2 Mitochondrial quality control

The myocardium and kidneys rely on mitochondria for ATP production to meet their high energy demands. Impaired mitochondrial metabolism reduces ATP synthesis, thereby impairing cardiac and renal function (14, 15). Exercise activates AMPK signaling and calcium transients to upregulate PGC-1α, the master regulator of mitochondrial biogenesis, and modulates the balance between mitochondrial fusion and fission, thereby preserving normal cellular function in cardiac and renal tissues. Additionally, exercise induces BNIP3-mediated mitophagy which eliminates dysfunctional mitochondria. These coordinated mechanisms restore mitochondrial integrity, improve cardiac ejection fraction and peak oxygen uptake, stabilize renal function, and enhance skeletal muscle oxidative capacity (16, 17).

### 2.3 Endoplasmic reticulum and oxidative stress modulation

Endoplasmic reticulum stress (ERS), triggered by hypoxia, calcium dyshomeostasis, or autophagic impairment, disrupts the nitric oxide/reactive oxygen species (NO/ROS) balance, thereby activating cascades implicated in cardiovascular disease and diabetes. Exercise intervention significantly downregulates the expression of key ERS proteins, such as PERK, IRE1α and ATF6, thereby alleviating endoplasmic reticulum stress. Coactivation of the metabolic regulatory factors PPARδ and PGC-1α represents another important mechanism that alleviates ERS through multiple pathways, including enhanced lipid metabolism, reduced oxidative stress, and regulated gene transcription (18, 19). While direct measurements (PERK, IRE1α, ATF6) in human exercise studies remains scarce, a pilot study (*n* = 16 elderly men) demonstrated that 12 weeks of resistance exercise training reduces ATF4 target genes (GADD45A, GADD34), implicating translational regulation of PERK signaling. However, the limited sample size precludes definitive conclusions regarding this mechanism in clinical populations (20).

Exercise exerts dose-dependent effects on oxidative stress. Moderate training induces beneficial hormesis, transiently elevating ROS to stimulate mitochondrial biogenesis and antioxidant enzyme expression (SOD, GPx), thereby enhancing muscle antioxidant capacity and improving cardiovascular function, insulin sensitivity, and chronic disease risk reduction. Chronic exercise activates the NRF2/ARE pathway, upregulating phase II detoxification enzymes (HO-1, NQO1) to maintain redox homeostasis. The regulation of oxidative stress confers several protective effects on the cardiovascular and renal systems. It maintains vascular endothelial function, reduces arterial stiffness, and enhances blood pressure regulation, thereby diminishing the risk of cardiovascular events such as myocardial infarction and stroke. Simultaneously, it mitigates renal oxidative damage and proteinuria, thereby decelerating the progression of CKD. However, excessive high-intensity exercise may surpass the compensatory threshold, exacerbating pathological stress responses. Therefore, individualized exercise prescriptions are essential for optimizing therapeutic efficacy and achieving sustainable health benefits (21, 22).

### 2.4 Inflammatory modulation

Chronic inflammation, a systemic low-grade state associated with aging, is a key precursor of CKM syndrome, inducing insulin resistance (IR), oxidative stress, arterial stiffness, and endothelial dysfunction (1). Exercise modulates inflammatory responses via multiple mechanisms. First, exercise suppresses pro-inflammatory mediators (TNF-α, IL-1β, MCP-1) and upregulates anti-inflammatory cytokines (IL-10, IL-1ra), thereby attenuating adipose tissue macrophage infiltration and improving insulin sensitivity. Second, exercise enhances skeletal muscle glucose uptake, mitigating hyperglycemia-induced advanced glycation end product (AGE) formation and attenuating inflammation and IR. These effects collectively mitigate cardiovascular events, postpone the deterioration of renal function, and improve fundamental abnormalities associated with metabolic syndrome, thereby representing novel targets for comprehensive CKM management (23).

## 3 Advances in exercise rehabilitation across CKM components

**Figure 2:**
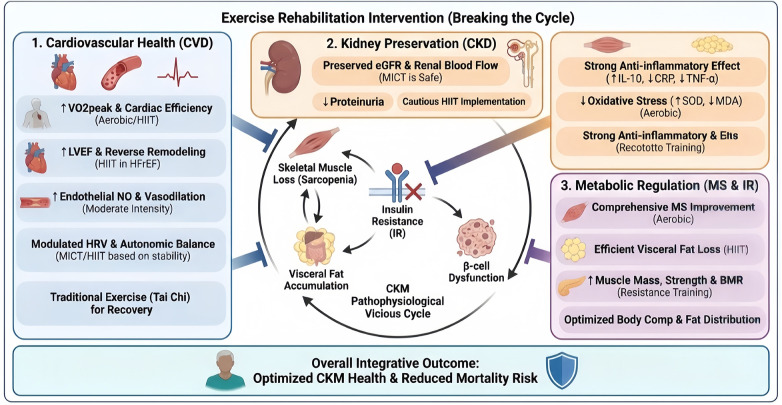
Integrative mechanisms of exercise rehabilitation in CKM syndrome (3, 31–33). CRP, C-reactive protein; HFrEF, heart failure with reduced ejection fraction; HIIT, high-intensity interval training; HRV, heart rate variability; LVEF, left ventricular ejection fraction; MICT, moderate-intensity continuous training; VO2peak, peak oxygen uptake.

Global population aging has positioned sarcopenia as a critical public health priority, with approximately 10% of older adults worldwide affected. Characterized by age-related declines in muscle mass and function (24), sarcopenia predisposes patients to adverse outcomes across all CKM components (25–27). The creatinine-to-body weight ratio (Cre/BW) reflects the interplay among adipose tissue, skeletal muscle, and lean body mass (28) and correlates strongly with chronic disease prevalence (27, 29). In older adults, elevated Cre/BW indicates relative fat deficiency, potentially increasing mortality from acute illness, whereas low Cre/BW reflects sarcopenic obesity, which compromises physiological reserve and increases mortality risk. Therefore, maintaining an optimal muscle mass is essential (28). Exercise modulates muscle and fat mass, optimizing body composition to maintain healthy levels and reduce health risks (30) (Figure 2).

### 3.1 Advances in exercise rehabilitation for cardiovascular diseases

The burden of CVD is strongly linked to cardiometabolic risk factors (6), which concurrently increases the prevalence of sarcopenia (25). Conversely, muscle loss is strongly associated with major cardiovascular conditions, including myocardial infarction, heart failure, atrial fibrillation, and atherosclerosis (31). Regular exercise increases muscle mass and reduces cardiovascular risk factors, thereby reversing this vicious cycle (3, 34).

#### 3.1.1 Effect of exercise rehabilitation on cardiopulmonary function

Peak oxygen uptake (VO2peak) is an independent predictor of CVD mortality (35). Aerobic exercise improves VO2peak, enhances myocardial contractility, improves cardiac pumping efficiency, increases LVEF in patients with heart failure (HF), and decreases left ventricular end-diastolic diameter (LVEDD), thereby alleviating cardiac burden. HIIT is superior to MICT in increasing aerobic capacity and reversing cardiac remodeling in patients with HFrEF (36). The REHAB-HF trial confirmed that in hospital initiation of early multimodal rehabilitation (balance, strength, mobility, endurance) improves physical function, frailty, and quality of life in high-risk acute decompensated heart failure patients. With 97% frailty prevalence and a mean 5 comorbidities (diabetes, obesity, hypertension, pulmonary, renal disease), this cohort mirrors CKM stage 4 phenotypes, supporting feasibility in this population (37). This trial provides a feasible rehabilitation plan for CKM stage 4 patients. In addition, traditional exercise rehabilitation has been gradually applied to the field of cardiac rehabilitation and has become an important supplement to modern cardiac rehabilitation systems (38). Traditional exercises (such as Tai Chi, Baduanjin, Wuqinxi, and Yijinjing) can enhance cardiac ejection function, increase the output per beat, reduce myocardial oxygen consumption, and improve cardiopulmonary function. They may serve as the cool-down phase of an exercise prescription, facilitating respiratory regulation and psychosomatic relaxation in patients (39).

#### 3.1.2 Effects of exercise rehabilitation on vascular endothelial function

Exercise enhances endothelial-type nitric oxide synthase activity and increases NO production, thereby improving vasodilatory functions. It can also increase the recruitment and mobilization of endothelial progenitor cells, thereby promoting neovascularization and restoration of endothelial function (34). Exercise-induced WSS at moderate intensity results in increased NO generation, whereas sustained high-intensity exercise-induced WSS produces excessive ROS, leading to decreased NO bioavailability and less improvement in endothelial function than moderate-intensity exercise (40). Li 2023 et al. (41) reached similar conclusions, that most forms of MICT and HIIT enhance endothelial function in both normal and impaired individuals, while continuous high-intensity exercise leads to endothelial dysfunction, further confirming the regulatory effect of exercise intensity on vascular endothelium.

#### 3.1.3 Effect of exercise rehabilitation on heart rate variability

HRV is an important indicator of the autonomic nervous system's function in regulating the heart and is usually associated with CVD risk (34). Both aerobic and resistance training modulate HRV in patients with CVD (42). In patients with chronic heart failure (35), a short intermittent HIIT protocol significantly increased HFnu% and improved vagal nerve tension, outperforming MICT. However, P. Eser 2022 et al. (43) observed opposite results in patients with recent acute ST-segment elevation myocardial infarction (STEMI): the traditional 4 × 4-minute HIIT protocol led to a decrease in HRV indicators, whereas MICT showed a beneficial trend. This difference stems from variations in the patient population and training intensity: recent STEMI patients are in an acute stress state, in whom HIIT tends to over-activate the sympathetic nerve, leading to deterioration of HRV, whereas the moderate stimulus of MICT better facilitates vagal recovery. In contrast, patients have progress beyond the acute phase and demonstrate greater tolerance to high-intensity training. Furthermore, short-interval passive recovery induces a “vagal training” effect, whereas long-interval active recovery results in sustained sympathetic activation and incomplete recovery. Consequently, MICT represents a safer option for patients with CKM stage 3–4 and acute cardiovascular events, whereas short-interval HIIT may be suitable for clinically stable patients with CKM stage 1–2. HRV can also help determine exercise intensity. HRV can guide the selection of exercise intensity. Non-invasive continuous monitoring based on wearable devices can dynamically adjust the exercise plan according to the autonomic nerve state: high-intensity training can be conducted when HRV is normal or high, while low-intensity activities or rest are recommended when it decreases to avoid the risk of overtraining (44).

### 3.2 Advances in exercise rehabilitation for chronic kidney disease

CKD, a key component of CKM (2), has become a public health issue that endangers human health. Physical inactivity is highly prevalent among patients with CKD because they fear that exercise might aggravate proteinuria and renal dysfunction (45). However, this sedentary behavior paradoxically increases all-cause mortality, particularly in end-stage renal disease (46). The KDIGO 2024 guidelines recommend that all patients should engage in at least 150 min of moderate-intensity exercise weekly as the primary component of renal rehabilitation, depending on cardiovascular and physical tolerance (32).

#### 3.2.1 Effect of exercise rehabilitation on kidney function

Although exercise influences renal hemodynamic function in healthy individuals, its impact on renal function across different intensities in patients with CKD remains inconclusive (47). A meta-analysis (48) showed that regular low-to-moderate intensity exercise had no negative impact on renal function in patients with CKD. MICT significantly improved blood creatinine, blood urea nitrogen, and 24-hour proteinuria levels in patients with CKD, while increasing the eGFR by promoting renal blood circulation, reducing oxidative stress, alleviating renal atherosclerosis, and lowering creatinine levels (5). These studies have proven that MICT is safe for patients with CKD. However, the effects of high-intensity exercise on renal function require further investigation. Early studies suggested potential renal damage, but recent evidence indicates that the risk only increases when it exceeds the lactate threshold (LT). As Kawakami et al. (45) noted, a single exercise session at LT intensity maintains renal blood flow (RBF) and eGFR without inducing any kidney injury. Using ultrasound to assess post-exercise renal hemodynamics, they found that neither MICT nor HIIT significantly affected RBF, blood flow velocity (BFV), and cross-sectional area (CSA), indicating that short-term HIIT does not reduce RBF and appears safe (45). These results align with findings by Hallan et al. (49), who reported that long-term HIIT reduces the risk of rapid eGFR decline by 25%. However, most existing studies are limited to healthy older adults, and direct evidence regarding HIIT in patients with CKD remains insufficient. Thus, the core issue is not whether HIIT is harmful or beneficial for patients with CKD, but the conditions, patients and forms in which HIIT can be safely and effectively implemented. Current evidence suggests that focusing solely on absolute exercise intensity is inadequate; instead, individualized intensity setting, professional supervision, and careful patient selection (such as those with stable early-stage CKD are crucial for renal safety). Future high-quality randomized controlled trials across different CKD stages are urgently needed to clarify the safety boundaries and optimal target populations for HIIT.

#### 3.2.2 Effect of exercise rehabilitation on inflammation and oxidative stress in CKD

Patients with CKD generally have a microinflammatory state and oxidative stress due to decreased renal function, immune dysfunction, and dialysis, conditions that progressively worsen as renal function decreases and, in turn, accelerate the deterioration of renal function (50). Meta-analyses (51) have demonstrated that exercise reduces inflammatory markers in CKD patients, and this effect is particularly evident for CRP in those undergoing dialysis. Compared with aerobic exercise, resistance training shows better anti-inflammatory effects, as it increases muscle mass, elevates anti-inflammatory marker IL-10, and upregulates TNF receptors, which inhibit TNF-α and reduce inflammation (52). Even a short intervention had a modulatory effect on the TNF-α levels. In summary, resistance training may be the primary exercise type for improving anti-inflammatory effects. Clinical research on the impact of exercise on oxidative stress in patients with CKD remains relatively limited. Small DM et al. (53) conducted a 12-month exercise intervention in patients with CKM stage 3–4 CKD, which resulted in an 11% increase in VO₂max and a reduction in BMI, confirming the safety of long-term exercise in this population. Notably, systemic oxidative stress markers remained unchanged in that study, likely due to the limited sample size and high baseline variability. Subsequent evidence from a recent meta-analysis has shown that aerobic exercise significantly improves oxidative stress profiles in patients with CKD, including increased superoxide dismutase (SOD) activity and decreased levels of malondialdehyde (MDA), F2-isoprostanes (F2-iso), and advanced oxidation protein products (AOPP), thereby enhancing antioxidant capacity and reducing oxidative stress end products, particularly in maintenance hemodialysis (MHD) patients (54).

### 3.3 Advances in exercise rehabilitation for metabolic syndrome

Metabolic Syndrome (MS) is an important risk factor for CKM (9), encompassing multiple metabolic abnormalities, such as hypertension, hyperglycemia, dyslipidemia, and central obesity, which contribute to the development of all subtypes of CVD (8) and play an important role in the pathophysiology of CKM (2). Exercise positively impacts all components of MS, and different exercise types optimize a patient’s body composition by regulating metabolic indicators and enhancing overall health (33).

#### 3.3.1 Effect of exercise across components of the metabolic syndrome

The effects of different exercise modalities on improving MS components of MS varies significantly. Aerobic exercise is the most effective in comprehensively improving core MS indicators, which can effectively improve key indicators such as waist circumference, TG, high-density lipoprotein cholesterol, fasting blood glucose, and blood pressure (55). Thus, aerobic exercise is the preferred rehabilitation strategy for patients with MS. In terms of exercise intensity, HIIT has more application value than MICT. HIIT improves efficiency and is a more feasible approach for managing overweight and obesity (56). Resistance training also exerts beneficial effects on MS. A recent review (57) indicated that resistance training improves all MS components augments muscle fiber number and cross-sectional area, and improves skeletal muscle functional capacity. Ding's (58) network meta-analysis identified optimal exercise doses for metabolic syndrome: HIIT at 561–672 METs-min/week reduced LDL-C and triglycerides, while benefits diminished beyond 1,500 METs-min/week, suggesting overtraining should be avoided. Traditional exercises are complementary to the treatment of MS, not only regulating glucose-lipid metabolism and lowering blood pressure, but also enhancing physical and mental health. Especially for middle-aged and elderly patients, traditional exercises have a soothing rhythm, slow and gentle movements, can adjust the speed and amplitude of the movements according to individual physical conditions, do not require complex equipment or specific venues, and the exercise process is accompanied by music and melody, making the practice process more pleasurable and relaxing, and are easier to promote and implement among the population (37, 59, 60).

#### 3.3.2 Exercise for the improvement of insulin resistance in metabolic syndrome

Skeletal muscles play a crucial role in the homeostatic regulation of systemic glucose-lipid metabolism. Muscle loss leads to decreased systemic glucose uptake and visceral fat accumulation, which triggers IR (27). In chronic insulin resistance, β-cell dysfunction accelerates the progression of MS to T2DM, significantly increasing the risk of vascular and renal diseases (8). Weight loss achieved through exercise is usually accompanied by a reduction in visceral fat, which contributes to improved IR (7). Notably, a recently published meta-analysis including 33 RCTs showed that exercise combined with nutritional strategies was superior to either modality alone, suggesting that dietary modification is essential for maintaining exercise-induced improvements in insulin resistance (61). Exercise-induced IR improvements without weight loss are reversible, with insulin sensitivity falling back to baseline levels once exercise is ceased (7). Different types of exercise have an improving effect on IR. Eight weeks of aerobic exercise lowered insulin levels and the HOMA-IR index, whereas 4 weeks of aerobic exercise had a less pronounced effect. This suggests that exercise duration is critical for improving IR (62). Differences in exercise intensity also have an impact. High-intensity exercise is more effective in reducing visceral fat and may improve IR more than moderate-intensity exercise. Compared to aerobic exercise, resistance training more markedly increases basal metabolic rate, enhances skeletal muscle mass and strength (63). The guidelines recommend that patients should engage in at least 150 min of moderate-intensity aerobic exercise per week, such as walking, jogging, Tai Chi, cycling, fitness dancing, and rhythmic gymnastics. RT, including lifting dumbbells, sit-ups, push-ups, weighted squats, and gluteal bridges, can be combined with aerobic exercise for better outcomes (37, 64).

## 4 Comprehensive effects of exercise rehabilitation in CKM

Given that obesity is the core driving factor of CKM, clinical and public health strategies should prioritize its prevention and management. Pharmacological or surgical weight reduction may serve as adjuncts; however, optimal drug targets remain undefined, weight regain after cessation is common, and long-term safety data are scarce (2). Exercise rehabilitation, by contrast, offers combined efficacy and safety, representing the most feasible intervention currently available.

**Figure 3:**
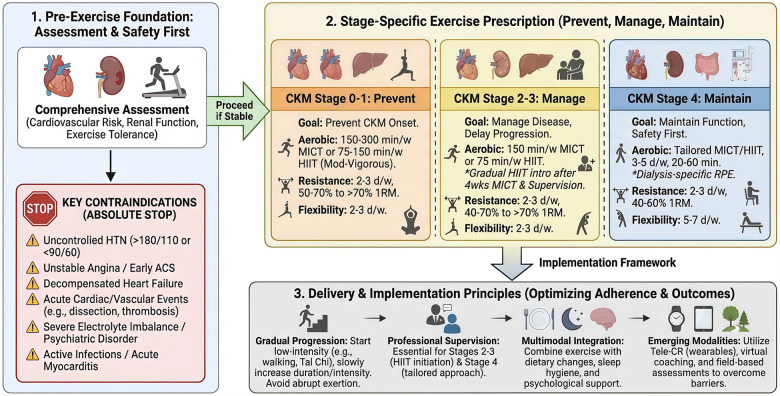
Integrative protocol: stage-specific exercise rehabilitation for CKM syndrome (3, 6, 7, 61, 64–66). Implementation suggestions: Pre-exercise cardiovascular, renal, and exercise capacity assessment is mandatory. For CKM stage 2–3, HIIT requires 4-week MICT preconditioning, clinical stability, and supervised titration; CAD patients need individualized risk stratification. CKM stage 4 necessitates customized exercise prescriptions based on cardiac status, dialysis regimen, and comorbidities, prioritizing safety and functional preservation. Initiate with low-intensity activities (e.g., walking, Tai Chi), progressing gradually in intensity, duration, and frequency. A multimodal lifestyle intervention integrating exercise, nutrition, sleep, and psychological management is recommended. Contraindications: uncontrolled hypertension (blood pressure >180/110 mmHg) or hypotension (<90/60 mmHg), early stage of acute coronary syndrome, unstable angina pectoris, decompensated heart failure, third-degree atrioventricular block, acute myocarditis or pericarditis, active infective endocarditis, acute aortic dissection, acute thrombotic phlebitis, deep vein thrombosis, severe electrolyte imbalance, severe psychiatric disorder, or inability to cooperate.

Based on the evidence, the exercise prescriptions for specific stages are summarized in Figure 3, as detailed in Supplementary Table S1 and Supplementary Table S2. The total weekly exercise doses for different exercise prescriptions are provided in Supplementary Table S2 (58, 65).

## 5 Limitations

Although this review synthesizes current evidence on exercise rehabilitation in CKM, several methodological and clinical limitations persist. First, mechanistic insights are predominantly derived from animal models, with a notable scarcity of high-quality human studies. While existing evidence suggests that exercise targets shared pathways involved in multi-organ dysregulation in CKM, it is crucial to emphasize that these mechanistic associations have not been causally established in humans. Future research should integrate methods such as tissue-specific gene editing and Mendelian randomization to elucidate the core mechanisms underlying the benefits of exercise. Second, we did not perform a systematic search or formal quality assessment of all included studies. Furthermore, exercise prescriptions across randomized trials and meta-analyses are highly heterogeneous, limiting cross-study comparability and precluding precise quantitative estimates for any endpoint. Research on individualized strategies remains scarce, as most studies do not stratify patients by CKM stage, comorbidity burden, or functional reserve, and they employ disparate outcome measures. These factors impede evidence synthesis and clinical translation. Finally, many studies continue to adopt a single-disease model, overlooking the multi-organ nature of CKM. High quality, stage specific evidence for exercise in advanced CKM is still lacking.

## 6 Conclusions and outlook

Exercise interventions play a crucial role throughout the entire course of CKM. CKM stage 1reduces body fat and visceral adiposity; CKM stage 2 improves endothelial function and optimizes glycemic-lipid control, thereby reducing metabolic risk factors; CKM stage 3 delays atherosclerosis progression while enhancing cardiac output and autonomic balance; and CKM stage 4 strengthens cardiac rehabilitation and reduces the risks of readmission and mortality (7). Consequently, exercise provides benefits at all stages of CKM.

## 7 Unresolved issues and research gaps

However, it still faces some challenges. Firstly, there is a lack of personalized exercise programs based on CKM staging. Most existing studies focus on single diseases or patients without staging, lacking standardized exercise recommendations by stage. Secondly, CKM emphasizes the joint intervention of cardiology and nephrology-endocrinology, but the integration of exercise rehabilitation is still insufficient. In clinical practice, physicians tend to rely on pharmacotherapy, which often hinders the formulation and implementation of exercise prescriptions due to the lack of support from interdisciplinary teams.

Going forward, it is necessary to explore precise exercise rehabilitation based on staging, establish a multidisciplinary collaboration team involving cardiology, nephrology and rehabilitation, and integrate exercise, medication, nutrition and psychological intervention to form a comprehensive care model. At the research level, large-scale randomized controlled trials targeting specific CKM stages should be conducted to standardize exercise prescription parameters and evaluation indicators. The focus should not be on simply debating the pros and cons of different exercise methods, but on establishing a process for formulating individualized exercise prescriptions based on precise assessment (including cardiovascular risk, renal function, exercise tolerance and autonomic nerve status). Wearable devices should be utilized to monitor real-time data and dynamically adjust exercise intensity to achieve safe and effective personalized rehabilitation.
