# Supplementary material for: Exercise rehabilitation in cardiovascular-kidney-metabolic syndrome: a narrative review
Source: Front Cardiovasc Med. 2026 Mar 27;13:1735431. doi: 10.3389/fcvm.2026.1735431 (PMC13065647; doi:10.3389/fcvm.2026.1735431)

Description of the literature search and screening process

This study employs a systematic literature search and screening strategy to ensure the comprehensive acquisition of existing evidence related to "cardiovascular-kidney-metabolic syndrome" (CKM) exercise rehabilitation. The flowchart is shown in Supplementary Figure S1. The specific process is as follows:

1. Database search:

(a) In six databases including PubMed, Embase, Web of Science, Cochrane Library, China National Knowledge Infrastructure (CNKI), and Wanfang Data, relevant literature published from January 2010 to December 2025 was retrieved.

(b) The search strategy was constructed by combining the key terms “exercise rehabilitation” and “CKM” and its components “(cardiovascular diseases, chronic kidney diseases, metabolic syndrome)” with free words.

2. De-duplication processing:

Import the search results from each database into the literature management software (such as EndNote), and the system will eliminate duplicate records.

3. Title and Abstract Preliminary Screening:

(a) Two researchers independently screened the titles and abstracts of the literature, and excluded the following types of documents;

(b)Studies not related to CKM or sports rehabilitation;

(c) Non-English literature (Due to resource limitations and considerations regarding

the quality of evidence, most of the CKM guidelines are published in English);

(d) Exceptional case: Traditional Chinese medicine exercises such as Tai Chi and Baduanjin, which have been included in the intervention studies, are significant supplements to CKM rehabilitation due to their cultural specificity, unique mechanisms, and being recommended by the WHO.

4. Full re-screening: For the screened literature, obtain and evaluate the full text, and exclude the following situations;

(a) The study did not involve any exercise intervention;

(b) The research subjects do not match (such as non-CKM-related populations);

(c) The data are incomplete or unable to extract valid information.

5. Supplementary Search and Evidence Expansion: Given the limited direct evidence of CKM, this study also included exercise intervention studies for its various components (cardiovascular diseases, chronic kidney diseases, metabolic syndrome) to infer the mechanism and clinical applicability. Manually search the references of the included literature and high-quality clinical guidelines (AHA 2023, ESC 2021, KDIGO 2024) to supplement potential related studies.

6. Final inclusion:

After going through the above process, a total of 67 documents were finally included, including:

(a) Direct evidence research on CKM.

(b) Study on CKM components.

(c) Research on Traditional Chinese Medical Exercises.

All the mechanisms described, evidence synthesized, and prescription recommendations included in the literature were used for the review.

Supplementary Figure S1

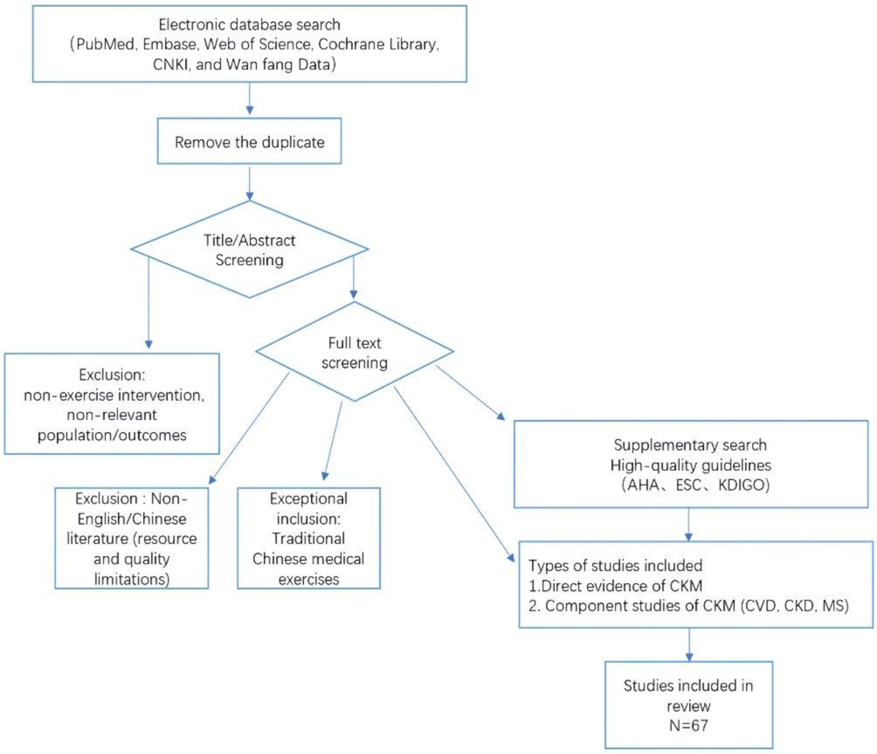

Supplement: Supplementary file 1 [file Datasheet1.pdf]
