# Supplementary material for: Exercise rehabilitation in cardiovascular-kidney-metabolic syndrome: a narrative review
Source: Front Cardiovasc Med. 2026 Mar 27;13:1735431. doi: 10.3389/fcvm.2026.1735431 (PMC13065647; doi:10.3389/fcvm.2026.1735431)
Supplement: Supplementary file 2 [file Datasheet2.pdf]

Supplementary Table 1. Exercise prescription for CKM

Stage		CKM stage 0-1	CKM stage 2-3	CKM stage 4
Characteristics		Metabolic risk predominant; no CVD/renal damage	Early CVD/renal damage	Severe CVD events or renal failure
Targets		Prevent CKM onset/progression	Manage subclinical disease; delay progression	Maintain function; reduce complications;
Aerobic Exercise	Frequency	150-300 min/w MICT (225-450 min for weight loss) or 75-150 min/w HIIT	150 min/w MICT or 75 min/w HIIT	MICT or HIIT 3-5 d/w, 20-60 min/d
	Intensity	RPE 11-13 (moderate)<br>RPE 15-17 (high)	RPE 11-13 (moderate)<br>RPE 15-17 (high)	RPE 11-13 (dialysis)<br>RPE 11-16 (non-dialysis)
	Types	Walking, swimming, jogging, cycling, rowing, climbing stairs, etc.		
Resistance Training	Frequency	2-3 d/w, 8-10 major groups, 10-15 reps, 2-4 sets, 2-3 min RI		2-3 d/w, 8-10 major groups, 8-15 reps, 1-3 sets, 2-3 min RI
	Intensity	50%-70% 1RM (mod)<br>>70% 1RM (vig)	40%-70% 1RM (mod)<br>>70% 1RM (vig)	40%-60% 1RM
	Types	Fixed weight machines, elastic bands, dumbbells, sandbags, bodyweight resistance, etc.		
Flexibility	Frequency	2-3 d/w, 20-30 min		5-7 d/w, ≥10 min/d
	Intensity	Stretch to mild tension （without pain）		
	Types	Yoga, square dancing, gymnastics, Tai Chi, Ba Duan Jin, balance training (high risk)		
METs-min/week		645-1440	525-1197	450-1197

d/w: days per week; reps: repetitions; mod: moderate intensity (50%-70% 1-RM); vig: vigorous intensity (70%-85% 1-RM); RI: rest interval.
