# Supplementary material for: Exercise rehabilitation in cardiovascular-kidney-metabolic syndrome: a narrative review
Source: Front Cardiovasc Med. 2026 Mar 27;13:1735431. doi: 10.3389/fcvm.2026.1735431 (PMC13065647; doi:10.3389/fcvm.2026.1735431)
Supplement: Supplementary file 3 [file Datasheet3.pdf]

Supplementary Table 2. The total weekly exercise dose for different exercise prescriptions

METs-min/week	Intensity	Frequency	Time	Type
450	mod（3METs）	5d/w	30min/d	Tai Chi、Yoga
525	vig（7METs）	3d/w	25min/d	HIIT
645	mod（4.3METs）	5d/w	30min/d	Walking
1050	vig (7METs)	5d/w	30min/d	HIIT
1197	mix（6.65METs）	3d/w	60min/d	CAREX
1290	mod（4.3Ts）	5d/w	60min/d	Walking
1440	mod（8METs）	3d/w	60min/d	Bicycling

1RM: One Repetition Maximum; CAREX: Combined Rsistance Exercise; MET: Metabolic Equivalent of Task; RPE: Rating of Percevied Exertion.

1. METs-min/week represents total weekly exercise dose, METs-min/week= Intensity (METs/min) × Duration (min/session) × Frequency (sessions/week).
2. METs values were derived from the 2024 Compendium of Physical Activities, with aerobic training coded as 02000 (Aerobic, general, 7.3 METs), HIIT as 02210(High intensity interval exercise, moderate effort resistance,7METs), resistance training as 02050 (weight lifting, free weights, moderate to vigorous effort, 6.0 METs), walking as17302 (Walking, for exercise, 2.5 to 3.5 mph (4.0 to 5.6 km/h), with ski poles, Nordic walking, level, moderate pace,4.3METs), bicycling as 01030 (Bicycling, 12-13.9 mph, leisure, moderate effort, 8METs) and yoga as 02155 (Yoga, Hot Yoga, 3 METs) .
3. CAREX METs values were calculated as the average of aerobic and resistance components: (7.3 + 6.0)/2 = 6.65 METs.
